# Quantification of Volumetric Bone Mineral Density of Proximal Femurs Using a Two-Compartment Model and Computed Tomography Images

**DOI:** 10.1155/2018/6284269

**Published:** 2018-02-27

**Authors:** Yan-Lin Liu, Jui-Ting Hsu, Tian-Yu Shih, Dmytro Luzhbin, Chun-Yuan Tu, Jay Wu

**Affiliations:** ^1^Department of Biomedical Imaging and Radiological Sciences, National Yang-Ming University, Taipei 11221, Taiwan; ^2^Institute of Nuclear Engineering and Science, National Tsing Hua University, Hsinchu 30013, Taiwan; ^3^School of Dentistry, College of Medicine, China Medical University, Taichung 40402, Taiwan; ^4^Department of Radiology, Cheng Ching Hospital, Chung Kang Branch, Taichung 40764, Taiwan; ^5^Department of Radiology, Mackay Memorial Hospital, Taipei 10449, Taiwan

## Abstract

**Objectives:**

Dual-energy X-ray absorptiometry (DXA) is frequently used to measure the areal bone mineral density (aBMD) in clinical practice. However, DXA measurements are affected by the bone thickness and the body size and are unable to indicate nonosseous areas within the trabecular bone. This study aims to quantify the volumetric bone mineral density (vBMD) using computed tomography (CT) images and the two-compartment model (TCM) methods.

**Methods:**

The TCM method was proposed and validated by dipotassium phosphate (K_2_HPO_4_) phantoms and a standard forearm phantom. 28 cases with DXA scans and pelvic CT scans acquired within six months were retrospectively collected. The vBMD calculated by TCM was compared with the aBMD obtained from DXA.

**Results:**

For the K_2_HPO_4_ phantoms with vBMD ranging from 0.135 to 0.467 g/cm^3^, the average difference between the real and calculated vBMD was 0.009 g/cm^3^ and the maximum difference was 0.019 g/cm^3^. For the standard forearm phantom with vBMD of 0.194, 0.103, and 0.054 g/cm^3^, the average differences between the real and calculated vBMD were 0.017, 0.014, and 0.011 g/cm^3^. In the clinical CT image validation, a good linear relationship between vBMD and aBMD was observed with the Pearson correlation coefficient of 0.920 (*p* < 0.01).

**Conclusions:**

The proposed TCM method in combination with the homemade cortical bone equivalent phantom provides accurate quantification and spatial distribution of bone mineral content.

## 1. Introduction

Osteoporosis is the microarchitectural deterioration of bone tissue [1]. Its prevalence increases with age and reaches as high as 40% for women after menopause [2]. The loss of bone mass compromises bone strength and subsequently increases fracture risks. Osteoporotic fractures are one of the important healthcare issues in the aging population because of excess morbidity and corresponding financial costs. In addition, the mortality significantly increases in the first year following proximal femur fractures [3]. Dual-energy X-ray absorptiometry (DXA) is currently used as the clinical standard to estimate the areal bone mineral density (aBMD) of proximal femurs for the diagnosis of osteoporosis. However, DXA is limited by the two-dimensional nature of projection radiography. The aBMD, defined as the bone mass divided by the projected bone area, is affected by the bone thickness and the body size and is unable to provide information on the spatial distribution of bone mineral content within the proximal femur.

Quantitative computed tomography (QCT) and peripheral quantitative computed tomography (pQCT) are CT-based techniques that use 360° projections around patients to reconstruct the attenuation coefficients of tissue. With a hydroxyapatite (HA) or dipotassium phosphate (K_2_HPO_4_) calibration phantom, the correlation between CT numbers and volumetric bone mineral density (vBMD), defined as the mineral mass divided by the bone volume, can be established, providing the assessment of patient's bone strength and fracture risks [4–6]. QCT was also used to measure femoral neck and shaft dimensions and to establish their relationships with age [7]. The results indicated that the average trabecular vBMD in the femoral neck was 22% lower in the older males, whereas the average aBMD obtained from DXA was only 4% lower.

Micro-CT with less than 1 *μ*m spatial resolution was used to investigate the microarchitectural properties of trabecular bones and animal specimens [8]. The texture indexes, such as homogeneity and fractal dimension, were frequently used to correlate them with the bone mineral density [9–11]. In addition, three-dimensional morphometric parameters were identified for the femoral head-neck samples [12]. Although micro-CT can be used to quantify the effect of pharmacological treatments of osteoporosis, bone biopsies can only be measured. The small field of view (FOV) is a major obstacle that prevents micro-CT from clinical quantification of the bone mineral density. High-resolution multidetector CT (MDCT) was adopted to perform the texture analysis of trabecular structures and predict the fracture risk of proximal femurs [13–15]. The results indicated that the cortical thickness performed best to predict the osteoporotic fracture in the femoral neck. Dental cone beam CT has a higher resolution than MDCT. It was used to evaluate the oral trabecular bone microarchitecture [16, 17] and the radiographic bone density based on the voxel values [18, 19]. However, dental cone beam CT is difficult to apply to the clinical practice for BMD quantification because of its image artifacts and small FOV for spine and hip measurements.

In the current study, a two-compartment model (TCM) method in combination with a homemade cortical bone equivalent phantom was applied to convert CT numbers to the bone volume fraction (BVF) and vBMD. K_2_HPO_4_ phantoms and a standard forearm phantom with different BMD sections were used to validate the proposed method. In addition, vBMD was estimated from clinical pelvic CT images of the femoral neck in 28 patients. The results were then compared with aBMD measurements obtained from clinical DXA scans to validate the accuracy of the TCM method.

## 2. Materials and Methods

### 2.1. Two-Compartment Model

Assuming that a mixture is composed of two substances, the CT number of the mixture in CT images can be calculated from the linear attenuation coefficients of the mixture and water as follows:

(1) CTNmix=μ−mixμ~H2O−1·1000,

(2) μ−mix=va·μ~a+1−va·μ~b,

where CTN_mix_ is the CT number of the mixture, ${\tilde{\mu }}_{H_{2}O}$ is the average attenuation coefficient of water under a specific X-ray energy spectrum, and ${\overset{-}{\mu }}_{mix}$ is the average linear attenuation coefficient of the mixture. ${\overset{-}{\mu }}_{mix}$ can be obtained using (2), in which *v*_a_ and (1 − *v*_a_) are the volume percentages of materials a and b, while ${\tilde{\mu }}_{a}$ and ${\tilde{\mu }}_{b}$ are the X-ray spectrum-weighted linear attenuation coefficients of materials a and b, respectively. The volume and weight percentages of material a can be calculated using

(3) va=CTNmix/1000+1−CTNb/1000+1CTNa/1000+1−CTNb/1000+1,

(4) wa=va·ρaρmix.

In (3), CTN_a_, CTN_b_, and CTN_mix_ are the CT numbers of materials a, b, and the mixture measured from CT images. The weight percentage of material a can be obtained from the volume percentage and the density ratio of material a to the mixture.

Human bone is composed of cortical bone and bone marrow in different proportions [20]. Based on (3) and (4), BVF and vBMD are calculated as follows:

(5) BVF=CTNmix−CTNmarCTNcor−CTNmar,vBMD=wcor·ρmix=vcor·ρcorρmixρmix=BVF·ρcor,

where CTN_cor_, CTN_mar_, and CTN_mix_ are the CT numbers of the cortical bone, bone marrow, and bone mixture obtained from CT images, respectively; and *w*_cor_ and *ρ*_cor_ are the weight percentage and the density of the cortical bone, respectively. In this study, a K_2_HPO_4_ aqueous solution was used as a substitute for the cortical bone and water for the bone marrow. Using the TCM method, we can convert CT images to BVF and vBMD distribution maps directly.

### 2.2. CT Scans of Bone Equivalent Phantoms

A cortical bone equivalent phantom with the vBMD of 0.533 g/cm^3^ was prepared with the K_2_HPO_4_ aqueous solution [21]. CT scans (SOMATOM Sensation 16, Siemens AG, Forchheim, Germany) were performed with peak tube voltages of 80, 100, and 120 kVp. A 30 mm × 30 mm region of interest (ROI) was drawn to calculate the average CT number and standard deviation for CTN_cor_. In addition, a water vial was scanned to acquire the average CT number and standard deviation for CTN_mar_. Seven different concentrations of K_2_HPO_4_ solutions from 0.135 to 0.467 g/cm^3^ were scanned. The CT image was converted to the vBMD map on a pixel-by-pixel basis for verification.

### 2.3. Validation Using a Standard Forearm Phantom

To further validate the accuracy of the TCM method, a standard forearm calibration phantom (QRM-EFP, GmbH, Moehrendorf, Germany) was used [22, 23]. The phantom is a 6 cm diameter and 6 cm height cylinder with both sides flattened by 1 cm. Four inserts with precisely 0.194, 0.103, 0.054, and 0-g/cm^3^ HA densities are labeled as sections (1) to (4) and are located in the left side of the phantom for the linearity check of CT scanners, while two inserts of water with the heights of 4.5 cm and 1.5 cm are located in the right side of the phantom. Each section is surrounded by a 1.2 mm thick wall with the HA density of 0.800 g/cm^3^ to simulate cortical bone. CT scans were performed at 80, 100, and 120 kVp, and the slice was acquired through the cortical wall. The CT image was converted to the vBMD map on a pixel-by-pixel basis. ROIs were selected in the center of the sections, and the average vBMD values and standard deviations were calculated. The accuracy of vBMD conversion using the TCM method was evaluated.

### 2.4. Validation Using Clinical CT Images

Patients who underwent DXA scans of proximal femurs for the diagnosis of osteoporosis and pelvic CT scans due to other clinical illnesses within six months were retrospectively collected, including 28 cases (17 males and 11 females). The average age of male subjects was 46.4 years (range: 30 to 69 years), whereas the average age of female subjects was 48.4 years (range: 34 to 59 years). No datasets were manually excluded from the analysis. The experimental protocols were approved by the Research Ethics Committee of Cheng Ching General Hospital and carried out in accordance with ICH-GCP guidelines.

For the DXA scans, a GE Lunar Prodigy system (GE Healthcare Lunar, Madison, WI, USA) was used to acquire both femurs. Automatic ROI analysis was performed and aBMD was measured using the standard hip protocol provided by the manufacturer. For the CT scans, the tube voltage of 120 kVp and automatic exposure control were used. Coronal images were reconstructed with the slice thickness of 3 mm. The slice with the maximum femoral neck area and its two adjacent slices were selected, and a volume of interest (VOI) was drawn over the femoral neck region. Segmentation of the bone and the surrounding soft tissue was based on the threshold of 250 Hounsfield Unit (HU) [24]. The average CT number of the femoral neck was converted to vBMD using the TCM method. The vBMD and aBMD were compared by the linear regression analysis. In addition, the relationship between patient's age and vBMD was evaluated for each gender. Pearson correlation coefficients between vBMD/aBMD and vBMD/age were calculated by SPSS version 19.0 (SPSS Inc., Chicago, IL, USA).

In order to evaluate the intra- and interobserver reliability of the TCM method, observer 1 performed another VOI selection on the pelvic CT images with a two-month period. In addition, another observer was recruited and performed the TCM method for the interobserver reliability test. The interclass coefficient (ICC) of model 3, type 1, and definition of consistency was analyzed. The results were interpreted based on Portney and Watkins's suggestion [25].

## 3. Results

### 3.1. CT Scans of Bone Equivalent Phantoms

**Figure 1:**
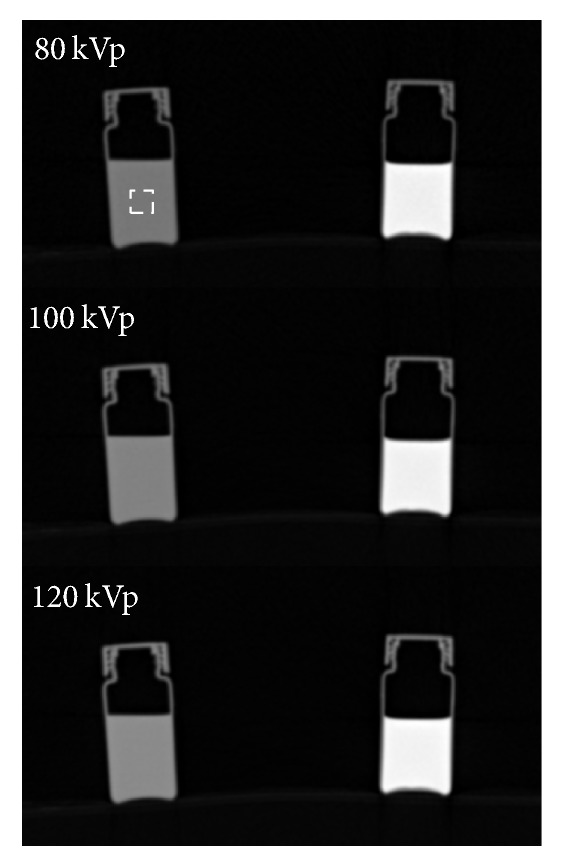
CT images of the water vial (left column) and the cortical bone equivalent phantom (right column) at 80, 100, and 120 kVp. A 30 mm × 30 mm ROI was drawn in the center of vials. The average CT numbers were taken as CTN_mar_ and CTN_cor_ for the TCM method.

Figure 1 shows the CT images of the water vial and the cortical bone equivalent phantom at 80, 100, and 120 kVp. The average CT numbers of water were −11.1 ± 30.3, 2.1 ± 23.7, and 8.7 ± 13.8, whereas those of the cortical bone equivalent phantom were 982.2 ± 41.4, 830.0 ± 27.2, and 739.5 ± 16.4 at 80, 100, and 120 kVp, respectively. The average CT numbers were taken as CTN_mar_ and CTN_cor_ in the TCM method for vBMD calculation.

### 3.2. Validation Using K_2_HPO_4_ Phantoms

**Figure 2:**
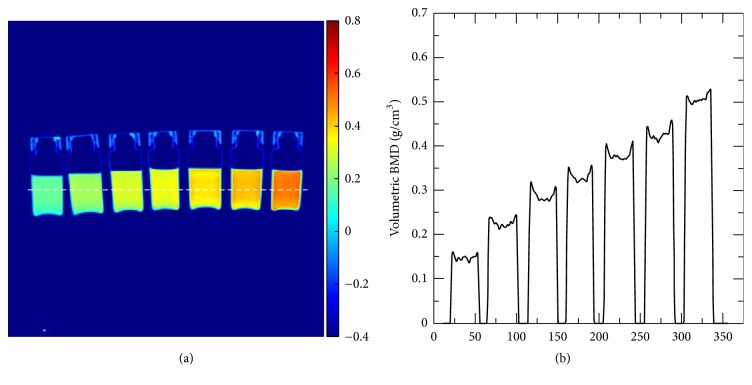
(a) vBMD map of K_2_HPO_4_ solutions with seven different concentrations from 0.135 to 0.467 g/cm^3^ from left to right calculated using the TCM method, and (b) the horizontal profile across the vials.

Figure 2 shows the vBMD map of the K_2_HPO_4_ solutions with seven different concentrations at 120 kVp. Their real vBMD values were 0.135, 0.202, 0.267, 0.300, 0.344, 0.401, and 0.467 g/cm^3^ from left to right. The calculated average vBMD values were 0.143, 0.215, 0.276, 0.306, 0.363, 0.407, and 0.468 g/cm^3^. The average difference was 0.009 g/cm^3^ and the maximum difference was 0.019 g/cm^3^. The horizontal profile of the vBMD distribution across the vials was depicted in Figure 2(b). Each vBMD level can be distinguished clearly.

### 3.3. Validation Using a Standard Forearm Phantom

**Figure 3:**
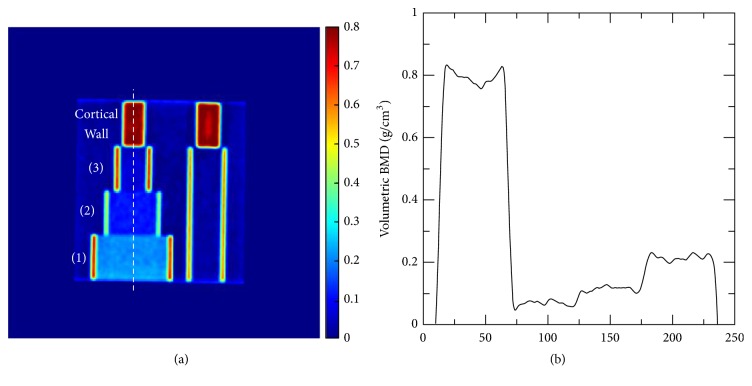
(a) vBMD map and (b) vertical profile of the standard forearm phantom converted from the CT image acquired at 120 kVp using the TCM method. The three sections and the cortical wall of the phantom can be clearly distinguished.

Figure 3 shows the vBMD map and the vertical profile of the standard forearm phantom converted from the CT image acquired at 120 kVp. The three sections and the cortical wall on the left side of the phantom can be clearly distinguished. The profile showed a slight underestimation in the center of the cortical wall area (Figure 3(b)).  Table 1 lists the real vBMD values of different sections and the average vBMD values calculated using the TCM method. The cortical wall consists of 0.800 g/cm^3^ vBMD, and sections (1) to (3) contain 0.194 to 0.054 g/cm^3^ bone density, which can be used to distinguish normal bone from osteopenia and osteoporosis. The average differences between the real and the calculated vBMD values for the three sections were 0.017, 0.014, and 0.011 g/cm^3^. The reason the calculated vBMD values were slightly larger than the real vBMD is that HA is used as the bone substitute in the forearm phantom and its effective atomic number is slightly higher than that of K_2_HPO_4_, which was used for the TCM calibration. In the cortical wall region, the calculated vBMD values were significantly underestimated under 80 and 100 kVp. This phenomenon could be caused by the beam-hardening effect in the CT image, which is prominent when low-energy X-ray photons pass through materials with high attenuation coefficients. The average differences between the real and the calculated vBMD values at 80, 100, and 120 kVp were 0.015, 0.013, and 0.011 g/cm^3^, respectively. The difference slightly decreased with increasing X-ray energy. Therefore, high kVp is preferred in the TCM method because of the low noise, low beam-hardening effect, and low atomic number dependence.

### 3.4. Validation Using Clinical CT Images

**Figure 4:**
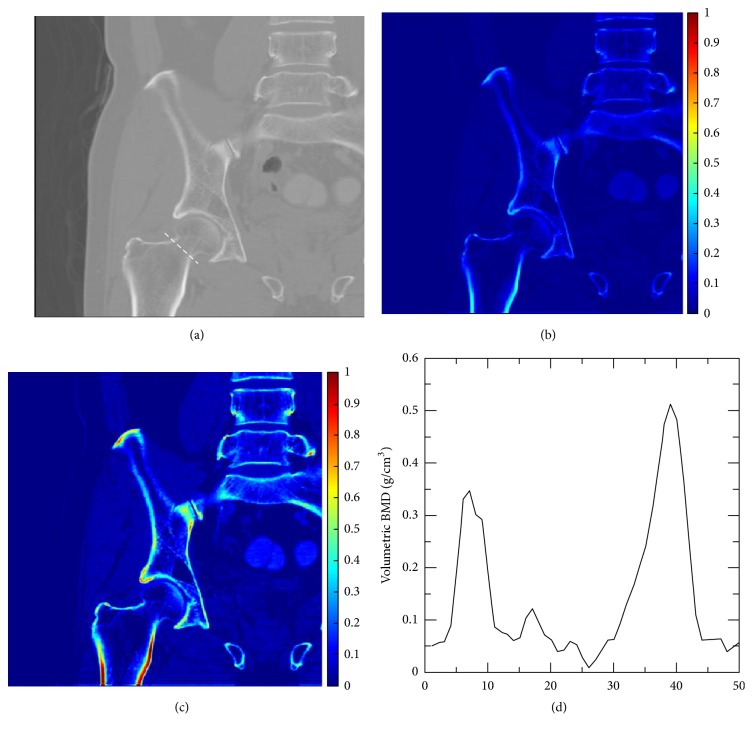
(a) Coronal CT image of a 53-year-old female patient, (b) corresponding BVF map, and (c) vBMD map calculated using the TCM method. (d) The profile of vBMD across the femoral neck showed a nonosseous area in the center of the trabecular bone.

Figure 4 shows a coronal slice of the proximal femur CT image at 120 kVp of a 53-year-old female subject and the corresponding BVF and vBMD maps calculated using the TCM method. The subject has been menopause, and no bone-related diseases or other medical histories have been reported. The surface of the femoral bone was covered by a layer of high-density cortical bone with less dense spongy bone inside. The vBMD value calculated by the TCM method was 0.123 g/cm^3^, whereas the aBMD obtained from DXA was 0.684 g/cm^2^. The profile across the femoral neck (Figure 4(d)) showed a nonosseous area within the femoral neck, indicating a high risk of osteoporotic fractures.

**Figure 5:**
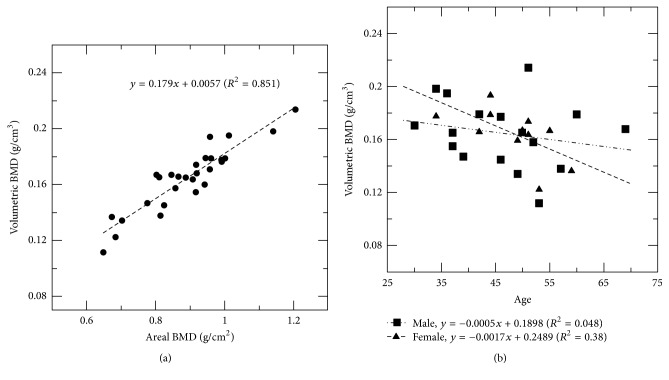
(a) Relationship between vBMD calculated using the TCM method and aBMD adopted from DXA, and (b) relationship between vBMD and age. The vBMD decreased with increasing age in both genders.

Figure 5(a) shows a scatter plot of vBMD calculated by the TCM method and aBMD adopted from DXA of 28 patients. There existed a good linear relationship between the two with the Pearson correlation coefficient of 0.920 (*p* < 0.01). The average aBMD was 0.892 ± 0.130 g/cm^2^ and the average vBMD was 0.166 ± 0.025 g/cm^3^. Among these patients, only one was diagnosed with osteopenia in the DXA report, and his vBMD value was also the lowest of all being 0.112 g/cm^3^. Both sensitivity and specificity are 100%.  Figure 5(b) shows the vBMD of the 28 patients as a function of age. The results demonstrated that vBMD decreased with increasing age in both genders. The Pearson correlation coefficient between vBMD and age for females was −0.615 (*p* < 0.05) and that for males was −0.220 (*p* = 0.397). Female subjects had a steeper drop in vBMD than male subjects. The aBMD data from DXA also showed a similar trend. For the intraobserver reliability of the TCM method, the ICC was 0.982 with 95% confidence interval between 0.961 and 0.992. For the interobserver reliability test, the ICC was 0.961 with 95% confidence interval between 0.918 and 0.982. Both results are interpreted as excellent in the level of reliability.

## 4. Discussions

Quantitative assessment of bone density can prevent osteopenia and osteoporosis. Currently, DXA is the most commonly used clinical method to measure aBMD. However, it is affected by the bone thickness and the body size. As a result, scoring systems, such as the *T*-score and *Z*-score, should be used to indicate the deviation from the mean for a specific population. A lower score suggests a higher chance of osteopenia and osteoporosis. Using the vBMD estimated by the TCM method allows us to perform an accurate quantification of bone mineral content, establish the spatial distribution of bone mass, and evaluate the hip structural properties. Moreover, vBMD estimated from CT images is not affected by the position and posture of patients, providing a comprehensive way to predict the femoral strength and fracture risk.

In this study, the vBMD value of the homemade cortical bone equivalent phantom is 0.533 g/cm^3^. The amount of HA in the QRM-EFP forearm phantom is up to 0.800 g/cm^3^. Estimating such high bone density could potentially undermine the assumptions of the TCM method. Therefore, if the accurate evaluation of vBMD of cortical bone is required, a higher concentration of K_2_HPO_4_ should be used for the cortical bone equivalent phantom. However, this leads to additional attenuation of low-energy X-ray photons, which generates the beam-hardening effect and interferes with the measurements. In this case, the use of high tube voltage and additional beam filtering is preferred to suppress the beam-hardening artifacts.

The American College of Radiology [26] recommends that osteoporosis is diagnosed when the three-dimensional bone density is less than 80 mg/cm^3^ and osteopenia be considered when the bone density is between 80 and 120 mg/cm^3^. Through the validation of K_2_HPO_4_ solutions, we proved that the proposed method is capable of diagnosing osteopenia with less than 5% error. The use of the standard forearm phantom with HA of 54 mg/cm^3^ has also proved that the TCM method can be used to diagnose osteoporosis with less than 10% error. If we further decrease the vBMD in the cortical bone equivalent phantom to half and use an equivalent material with the atomic number and density similar to those of real bone, the accuracy of estimating low vBMD values could be increased.

In the validation of clinical pelvic CT images, vBMD shows a decreasing trend with increasing age. The trend is more pronounced for females with a vBMD loss rate of 0.898%/year than for males with that of 0.290%/year. Several studies addressed the BMD changes as a function of age in both genders using DXA. Ardawi et al. [27] reported the BMD changes of 0.92%/year for females and 0.46%/year for males in Saudi Arabia, while Cui et al. [28] reported 0.91%/year and 0.50%/year for females and males in Korea, respectively. Our results for females match them very well; however, the fitting results for males are slightly lower than their outcome due to relatively large fitting errors.

The TCM method with CT images can also be used to calculate vBMD of the lumbar spine, forearm, wrist, heel, and so forth. When performing TCM measurements, the tube current of CT scans should be minimized by using automatic tube current modulation techniques to reduce the radiation dose delivered to patients, and the high tube voltage should be applied to increase the accuracy of bone density estimation. In addition, the TCM method can be performed along with other routine CT examinations to achieve the purpose of preventing osteoporosis. Future investigations should explore the use of the TCM method with low dose CT scans.

## 5. Conclusion

In the current study, we proposed the use of the TCM method to calculate BVF and vBMD from CT images. Through the validation of the K_2_HPO_4_ bone equivalent phantoms and the standard forearm phantom, the proposed method demonstrates accurate bone mineral quantification without the need of a specific QCT phantom under the couch. Through the validation of clinical CT images, the resultant vBMD of proximal femurs has a good linear relationship with the aBMD obtained from DXA. In summary, the TCM method in combination with the homemade cortical bone equivalent phantom can provide accurate quantification and spatial distribution of bone mineral content.

## Figures and Tables

**Table 1:** Comparison between real vBMD values and calculated vBMD values of different BMD sections using the TCM method at 80, 100, and 120 kVp. The *p* values in parentheses were calculated by comparing to the real vBMD values.

Section	vBMD (g/cm^3^)	80 kVp	100 kVp	120 kVp
(1)	0.194	0.215 ± 0.08 (0.161)	0.207 ± 0.04 (0.086)	0.210 ± 0.07 (0.221)
(2)	0.103	0.118 ± 0.08 (0.313)	0.117 ± 0.05 (0.138)	0.115 ± 0.06 (0.401)
(3)	0.054	0.069 ± 0.07 (0.250)	0.065 ± 0.09 (0.509)	0.061 ± 0.07 (0.588)
Cortical wall	0.800	0.790 ± 0.014 (0.001)	0.785 ± 0.012 (0.001)	0.789 ± 0.04 (0.143)
